# Using Social Network Analysis to Visualize Networks in American Indian Health Research

**DOI:** 10.33596/coll.22

**Published:** 2019-01-22

**Authors:** Jessica D. Hanson, Morgan E. Nelson, Abby Martin, Lindsay Erickson, Susan E. Puumala, Melissa Buffalo, DenYelle Baete Kenyon

**Affiliations:** 1 Population Health Group, Sanford Research, Sioux Falls, SD, US; 2 Avera Research Institute, Sioux Falls, SD, US; 3 Department of Mathematics, Augustana University, Sioux Falls, SD, US; 4 HDR, Inc., Omaha, NE, US

**Keywords:** Social network analysis, American Indian, research infrastructure

## Abstract

The Collaborative Research Center for American Indian Health (CRCAIH) is a transdisciplinary, collaborative center focused on building American Indian tribal research infrastructure. Funded by the National Institute of Minority Health and Health Disparities in 2012, it was created as a platform to join tribal communities and researchers in South Dakota, North Dakota, and Minnesota to develop research infrastructure and stimulate research in American Indian health. The CRCAIH infrastructure has created a large network of transdisciplinary research partnerships. To understand the initial development of the CRCAIH network and understand the broader impact it has had on American Indian and Alaska Native health research, CRCAIH undertook a network analysis based on publications by collaborators working with and within CRCAIH. The network analysis showed how far the CRCAIH network went in a short period of time to create a platform for networking to build collaborations and further stimulate research with American Indian communities.

## Background

Adequate funding is a common barrier to health research, but that is especially true for some states in the Upper Midwest of the United States. For example, North Dakota and South Dakota are among the eight lowest funded states in terms of federal grant dollars, receiving an average of $17.03/person, compared to the eight top funded states, such as California, Massachusetts, and New York, which receive an average of $132.87/person (NIH Research Portfolio Online Reporting Tools, 2015; U.S. Census Bureau, 2015). Funding for research is increasingly significant when considering the importance of adequate dollars in combating health disparities. For example, the low research funding in North Dakota and South Dakota is especially concerning considering the high proportion of American Indians living in this area (5.4% in North Dakota and 8.9% in South Dakota) (U.S. Census Bureau, 2015).

While the American Indian population is the smallest racial or ethnic group in the United States, it carries the largest burden of health risk factors and chronic disease (Jones, 2006; Norris, Vines, & Heffel, 2012; U.S. Census Bureau, 2015). These disparities include higher rates of diabetes, heart disease, liver disease, and mental health problems (Blackwell, Lucas, & Clarke, 2014; Jernigan, Duran, Ahn, & Winkleby, 2010; Sarche & Spicer, 2008). Health disparities are further exacerbated by poor socioeconomic factors including poverty and high unemployment in many American Indian communities (Macartney, Bishaw, & Fontenot, 2013; Ogunwole, Drewery, & Rios-Vargas, 2012; Sarche & Spicer, 2008; U.S. Bureau of Labor Statistics, 2016). These factors, along with availability of reliable healthcare, create barriers to health equity (Artiga, Arguello, & Duckett, 2013) and therefore create many areas in which health disparities research is needed.

### The Collaborative Research Center for American Indian Health

In 2012, the Collaborative Research Center for American Indian Health (CRCAIH) was established via a National Institutes of Health grant. The goal of CRCAIH is to enhance social determinants of health research in the American Indian population through collaboration with tribal communities and health researchers in South Dakota, North Dakota, and Minnesota. CRCAIH is composed of partners including tribal nations, communities, academic institutions, and health care entities, brought together on the common goal of reducing American Indian health disparities (Elliott et al., 2015). CRCAIH has been successful in building research infrastructure by collaborating with seven tribal partners across the tri-state region, funding three large-scale research projects and 15 pilot grants, and establishing several cores, including methodology/data analysis, regulatory, cultural ethics, and community engagement, to assist in the research process. This infrastructure has created a large network of transdisciplinary research partnerships.

### Social Network Analysis

There are many ways to measure the success of CRCAIH in its efforts to enhance research and develop new collaborations. One important way is to assess the expansive network of researchers from across the world that have emerged through the funding of CRCAIH. Social network analysis is a valuable tool to examine the initial network developed by CRCAIH researchers. Social network analysis is a way to visualize relationships to find where knowledge is being created and where it can be built further (Cross, Parker, & Borgatti, 2002). It can be used to improve collaboration, knowledge creation, and knowledge transfer in organization settings such as CRCAIH (Cross et al., 2002). This is especially beneficial to CRCAIH and other groups working on research in American Indian health since it is a tight-knit field that works with a relatively small population. Therefore, it is only through collaboration that researchers and communities can share resources to stimulate and further develop their research programs.

One of the goals of CRCAIH was to increase transdisciplinary collaborations for research in American Indian health. Therefore, the purpose of this paper is to highlight the connectivity of those involved with the CRCAIH project, specifically by using an initial analysis on the network of collaborations based on peer-reviewed publications funded through CRCAIH. This provides a preliminary investigation of collaboration and scientific productivity among these collaborations.

Through social network analysis, we will show how the CRCAIH network provided a hub for networking to build collaborations and further stimulate research.

## Methods

### Data Collection

Our main outcome was to determine the network of researchers connected through CRCAIH specifically by examining peer-reviewed publications on American Indian health research published between October 1, 2012 (when CRCAIH began) and June 30, 2016. The starting point for network creation was CRCAIH-funded publications published or accepted before June 2016, with additional criterion that first authors were all CRCAIH-funded researchers and the content focused on American Indian health disparities research. We then looked at all co-authors of the first authors of these CRCAIH-funded publications and their *other*, non-CRCAIH funded publications to extend the network to four degrees of separation from the center. We obtained CRCAIH-funded publications through our CRCAIH progress reports and then obtained other manuscript information from publicly available scholarly databases including PubMed, Google Scholar, and local university library resources. American Indian health publications included in our analysis had to be published or accepted for publication in a peer-review journal and include American Indian (or Native American, etc.) in the title, abstract, or keywords.

Data collected from each publication included author name, affiliation, location, email address, and publication citation information. Authors’ organizations were categorized into affiliation categories including: American Indian medical, American Indian based research, academic (non-American Indian specific), government, industry, medical (non-American Indian specific), research (non-American Indian specific), tribal college, and other tribal affiliated institutions (including tribal health boards, health services, and governments). Publications were coded into the following categories: community health/interventions, education, epidemiology/health disparities, ethics/regulatory/policy, genomics/metabolomics, and research methodology. The community health/interventions and epidemiology/health disparities categories were differentiated by the former describing community outcomes, interventions, and evaluations (community health/interventions) and the latter investigating differences in prevalence, incidence, and outcomes by various factors (epidemiology/health disparities). The ethics/regulatory/policy category contained publications on anything that informed health policy, research regulation, and factors that should be considered in healthcare or research including the American Indian perspective on health and research.

### Social Network Analysis

The first stage in the process of analyzing the network of collaborators was to develop and launch an online tool to view the entire network, as well as search authors and their personal network. To create meaningful visualizations based on collaborations, we used graph theory, the mathematical field of network structure and its analysis. We created two types of network graphs: a full graph visualization and individual author graphs showing each author’s first-degree collaborators. In this application, a vertex represents an author, an edge represents a collaboration between two authors, and the weight of the edge represents the number of papers crediting both as authors. For specific information on the way data was rendered into graphs, transitioned to online, and analyzed in Gephi, please see Appendix.

We next focused on computing the Small-World Index (Neal, 2017) to determine how intimately the authors are related through publications. The Small-World Index (SWI), which is a double-graph normalized index using random and lattice reference graphs, ranges from zero to one, where a value near one indicates a small-world network. The SWI measures, in our case, the degree to which the authors work within groups versus how groups of authors (grouped via shared publication) work with other groups of authors.

## Results

**Figure 1:**
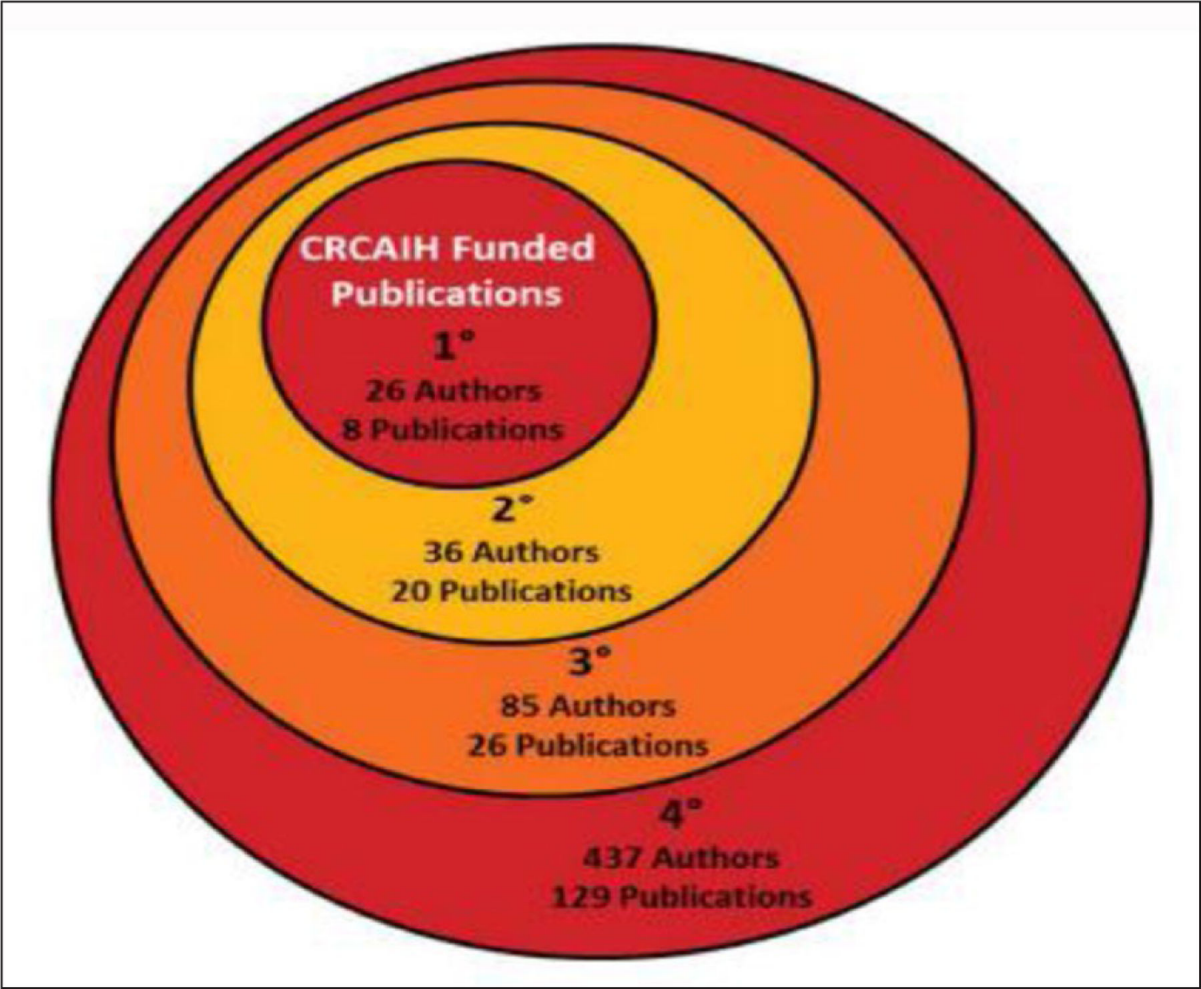
Breakdown of CRCAIH-linked Authors and Publications by Degree of Separation.

**Figure 2:**
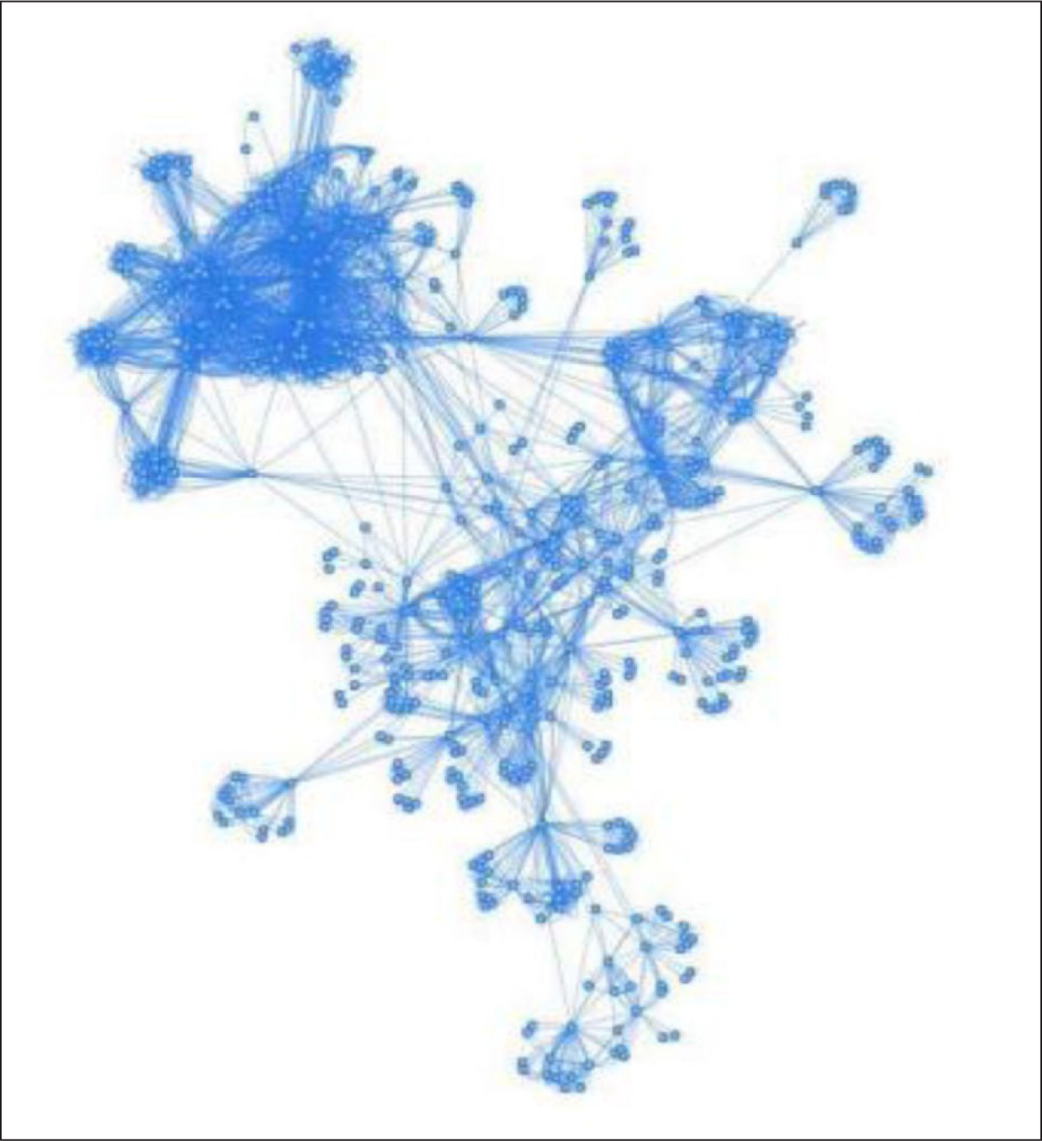
Network of CRCAIH-linked Researchers.

The CRCAIH-based network consisted of 584 authors from a variety of institutions. See Figure 1 for a breakdown of authors and publications by degree of separation, similar to Stanley Milgram’s famous “six degrees of separation,” which purports that the diameter of a collaboration network is six. Here individual people are the vertices and shared variables (publications in this case) are the edges. Figure 2 shows the expanse of the network that stems out from the CRCAIH researchers. In our graph of 584 authors, there are 170,236 possible pairs of undirected nodes, or connections between any two authors. In order to ensure connectedness, two isolated authors were removed from the graph, resulting in a total of 582 authors. There were several key authors that “bridged” between groups of people through their mutual work. Once a researcher collaborated with one of these key authors, they then became connected to a larger group of researchers with similar interests.

Table 1 outlines the authors by organizational affiliation, with the majority coming from non-American Indian specific groups such as research and academic institutions. Of note, is that 15% of authors were affiliated with American Indian-focused establishments, highlighting the potential to expand research and collaborations to some specific groups such as tribal colleges and tribally run organizations, even if the focus of those organizations is not research. Only 11.5% (n = 67) of the authors come from the states that CRCAIH focused its efforts on (South Dakota, North Dakota, and Minnesota), with others from states such as New York and Massachusetts. Besides traditional academic institutions, representatives also came from government organizations such as the National Institutes of Health. Nearly all the authors (97%) were based in the United States, while the remaining 3% (n = 20) of the authors were international from countries such as the United Kingdom, Germany, and New Zealand.

A total of 183 publications came from the 582 authors stemming from the CRCAIH network. While many of the CRCAIH staff, faculty, and research projects are focused in the social sciences and public health, the publication topics ran the gamut and represented a wide spectrum of content. See Table 2. While the most cited topic was epidemiology and/or health disparities, not surprising given the focus of CRCAIH, publications also included ethics and regulatory policy, research methodology, genomics, and education. A total of 22% of the publications in this network were dedicated to the ethics and regulation of research, and developing research methodology that accommodates the American Indian population’s needs and wants for research being done in their communities. This variety of topics is in direct parallel to the wide range of research interests expressed by CRCAIH tribal partners and points to the need for tribal research that goes beyond a focus on health disparities and intervention-based research.

**Figure 3:**
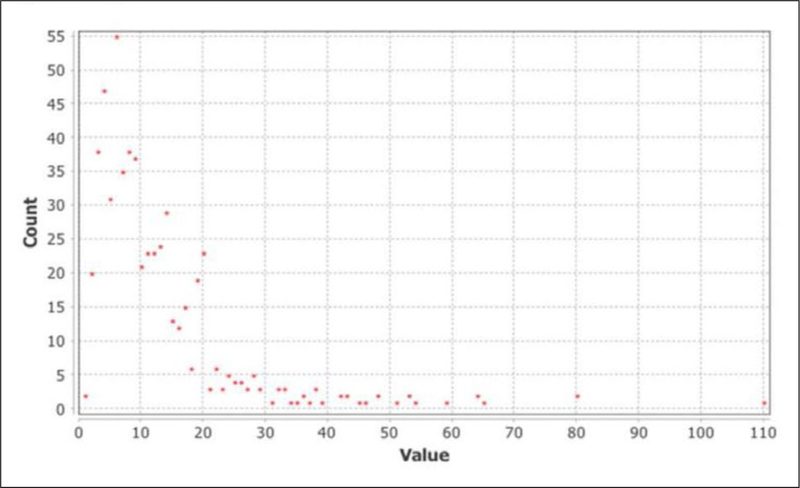
Degree Distribution.

We measured degree distribution, weighted degree distribution, and clustering. First, the degree distribution plotted the frequency of degrees (number of shared publications) for each vertex (author) of the graph. We rendered the CRCAIH network graph in such a way that authors who share multiple publications have the multi-edge suppressed to a single weighted edge. In this way, the vertex degree represents the total number of collaborators an author has with another author in the network. The average degree was 12.512, meaning that on average, an author co-authored with about 12 other authors in the network. See Figure 3. We found the average path length to be 3.879 (Brandes, 2001), meaning that on average, most authors are no more than 4 publications apart from any other author. Comparing to the “Six Degrees of Separation,” any given author is linked to any other author via four publications, indicating a rather tight-knit community.

Next, the weighted degree distribution plots the frequency of each vertex degree with the weight of each edge (as measured by the number of publications two authors co-authored) accounted for in the distribution. In the CRCAIH network, the weighted degree represents simultaneously the number of shared publications, as well as co-authorship. The average weighted degree in the graph was 16.869, meaning that on average, an author published nearly 17 different articles relating to the CRCAIH network.

To compute the SWI, we generated an Erdos-Renyi random graph on 582 vertices with an unweighted, undirected edge probability of 0.01767 to mimic that of our CRCAIH network. We found the average path length (APL_R_) of this random graph to be 2.781 and the clustering coefficient (CC_R_) to be 0.023. We also generated a random lattice graph on 582 vertices with an average degree of 12 and a beta value of 0.01767 to match that of our graph and the Erdos-Renyi random graph and CRCAIH network graph. For the lattice graph, we found APL_L_= 5.8498 and CC_L_= 0.5757576. For CRCAIH, we found average path length APL_A_ = 3.879 and average clustering coefficient CC_A_ = 0.868 (Latapy, 2008). Thus, our SWI was computed to be 0.98173. Interpreted according to the work of Neal (2017), an SWI near one indicates a strong prevalence of the small world effect. In our CRCAIH network, this indicates that communities collaborate with one another quite frequently.

## Discussion

### CRCAIH’s Reach

This network analysis shows the fruition of CRCAIH’s aim to create a platform with which to bring together tribal communities and health researchers of many disciplines to combat American Indian health disparities. The goal of the initial CRCAIH grant was expanded to other networks of researchers and touched on topics beyond health disparities into other areas tribal communities deemed important, such as policy, issues with research regulation, and how to develop research in genomics. There were several key researchers that pushed out work with American Indians to other groups of collaborators, further connecting academics and community researchers from across the world with a common goal of delving into American Indian research. The network analysis showed how far the CRCAIH network went in a short period of time to create a platform for networking to build collaborations and further stimulate research with American Indian communities. While this is just a snapshot that shows a certain level of collaboration between authors, it also highlights the potential for these types of large-scale collaborations to expand the breadth of health disparities research.

Along with this, our results demonstrated CRCAIH has successful links even beyond regional ones, with connections with researchers in diverse community, academic, and government institutions. There are representatives from government groups, such as the National Institutes of Health (NIH) and the Centers for Disease Control (CDC). Concurrently, academic institutions were well represented, such as Dine College and University of Washington, along with tribal nation research groups, such as the Center for Native and Pacific Health Disparities Research and Southcentral Foundation in Alaska. Finally, we showed that the CRCAIH social network included large corporations and foundations, including Pfizer Inc., tribal health institutions such as the Yukon-Kuskokwim Health Cooperation, and the Chickasaw Nation Department of Health. Many of these institutions have active collaborations, but more effort should be directed into continuing to strengthen these collaborations and building new connections with each other and others outside the network. Indeed, these results show the potential for CRCAIH to be viewed not only as a regional center, but as a national-level organization.

### Promising Methodology

Our study used social network analysis to describe these current networks, and there is potential to stimulate further collaborations by creating an interactive tool online that shows who is connected to whom and where potential collaborations could be built. This social network tool can be used to find the collaborators with which someone is already connected and see to whom someone can connect to build collaboration networks. These results highlight the importance of identifying and utilizing key researchers that are connectors to several successful groups of people. These key connectors can assist in building new collaborations through identifying and introducing researcher to potential collaborators or community members.

### Future Directions

Continuing CRCAIH’s goal of creating a platform for American Indian transdisciplinary research to bring together multidisciplinary researchers, tribal leaders, and community experts who share common interests in addressing American Indian health disparities is ideal. Looking back at the results and the goal of CRCAIH, only 11.5% of authors in our analysis focused their efforts within SD, ND, and MN, the states that comprise CRCAIH. However, these three states alone consist of 23 federally recognized tribal nations, each with their own unique interests and abilities to engage and conduct research. The 584 authors serve as the foundation for extending this established research network with American Indian communities. The social network created by CRCAIH provides a platform for research to combine various academic disciplines to be more effective in addressing complex health outcomes. The work initiated by the CRCAIH team and their partners goes beyond one discipline and has the potential to further incorporate culture, history, and Indigenous methodologies. CRCAIH represented well the interconnectedness of those relationships and the agenda from a tribe itself. A research network such as this is an opportunity for direct collaboration and continual dialogue, as well as broadening connections to improve American Indian health.

Of the 23 federally recognized tribes within the three states, CRCAIH has worked directly with seven, and is currently working with five to establish research infrastructure. Examples of the research infrastructure built are research review boards, tribal research codes, and research review policies and procedures, all of which have been approved by Tribal Councils. CRCAIH created a model platform that is both researcher and community inclusive, which fosters and builds trust, and that lays the groundwork in establishing relationships that are equitable and respectful of tribal sovereignty. CRCAIH continues to be a regional clearinghouse, acting as a repository of information for organizations, both tribal communities and research-intensive, as well as holding an annual in-person summit to highlight the strong work across the U.S. in American Indian health research. Because CRCAIH’s focus on the social determinants of health research is broad, it can continue to be a magnet for those interested in collaborating to improve American Indian health in a variety of topic areas, which is critical in a small field/low population dense region such as the Northern Plains. This is underscored by the fact that almost a quarter (22%) of the publications in the network were on research ethics, one of CRCAIH’s strengths in adding to the research literature, which is an important underpinning of all types of research with tribal nations. A future direction to continue to build the CRCAIH network is to do targeted matchmaking of researchers with tribal partners, based on researchers’ expertise and tribal partners’ research priorities. For example, we could look at the expertise of those key authors who bridge several groups of people and match them with tribal organizations.

In sum, we set out to demonstrate the breadth and depth of CRCAIH’s collaborations through a social network analysis of peer-reviewed publications. This is only one way to demonstrate partnership links, as often partnerships do not result in peer-reviewed publication. However, we posit it is a proxy of well-developed, high functioning research partnerships that demonstrate the impact of research funding and years of collaboration.

## Figures and Tables

**Table 1:** Authors Categorized by Affiliation.

Category	n (%)
**American Indian Specific Institutions**	**88 (15.1)**
American Indian Medical	6 (1.0)
American Indian Based Research	27 (4.6)
Tribal College	11 (1.9)
Other Tribal Institutions	44 (7.6)
**Non-American Indian Specific Institutions**	**495 (84.9)**
Academic	369 (63.3)
Government	31 (5.3)
Industry	5 (0.9)
Medical	64 (11.0)
Research	26 (4.5)

**Table 2:** Publications Categorized by Content.

Category	n (%)
Community Health/Interventions	43 (23.5)
Epidemiology/Health Disparities	70 (38.3)
Ethics/Regulatory/Policy	26 (14.2)
Research Methodology	15 (8.2)
Genomics/Metabolomics	26 (14.2)
Education	3 (1.6)

## Appendix

Using Java, we first wrote a program to represent a visualization of the entire network of authors, as well as individual graphs representing any one author. An adjacency matrix was used to transition the data from its original form into a structure that easily lends itself to the creation of the graphs used in the online rendering of the network. An adjacency matrix is an (*n* × *n*)-matrix such that the (i, j)^th^-entry of the matrix consists of the integer amount of publications between author *i* and author *j*. In this way, the matrix took into account the number collaborations between authors within a network represented for the weight of the edges. Due to the need for individual graphs, a simple overall adjacency matrix encompassing all authors and publications did not appear to be the best way to store this data. Instead, an ‘Author class’ and ‘Paper class’ were implemented in Java to create a modified adjacency matrix. The matrices were created by finding the collaborations between authors by incrementing through the paper list and then iterating through the author list associated with the current paper. For each author, all the collaborators on the paper were added to the list of that author’s collaborators and their associated number of collaborations was either incremented if they already existed on the list or set to one if the author was a new edition. This process created each author’s row of the adjacency matrix, and continued for all authors and all publications creating the full adjacency matrix.

To create the individual author graphs, the respective author’s row of the adjacency matrix was used to identify that author’s collaborators and number of collaborations. An edge was added to connect the original author with all their coauthors in the graph and weighted by the number of collaborations. For each pair of coauthors, each author’s row of the adjacency matrix was searched for only the other coauthors, and those collaborations were added as weighted edges to the original author’s graph. This way, all the authors that appeared in the visualization had collaborated with the central author, and all interconnections within this network were represented. This was performed for all authors within the network.

To create the full network graph, the full adjacency matrix was used. Edges were used to connect all collaborators and weighted by the number of collaborations. Authors were represented by dots in this graph whereas by their names in the individual graphs. A number of metrics were used to analyze the characteristics of the network. The metrics for the CRCAIH network and the Erdos-Renyi random graph were measured in the graph analysis program Gephi. In order to convert the database to a readable table for Gephi, we wrote an algorithm in R to take the adjacency matrices and render them as a double-column list. Input is a graph with the “from” column representing a paperID and the “to” column representing an authorID. The goal was to link all authors in a paper in graph form. Because ID’s were consistent across the file, authors that collaborate on multiple papers were then linked. The random lattice graph was constructed and analyzed in Python.
